# A case-control study of the *HER2 *Ile655Val polymorphism in relation to risk of invasive breast cancer

**DOI:** 10.1186/bcr1004

**Published:** 2005-03-11

**Authors:** Stephanie E Nelson, Michael N Gould, John M Hampton, Amy Trentham-Dietz

**Affiliations:** 1 McArdle Laboratory for Cancer Research, University of Wisconsin, Madison, Wisconsin, USA; 2 Department of Oncology, University of Wisconsin, Madison, Wisconsin, USA; 3 University of Wisconsin Comprehensive Cancer Center, Madison, Wisconsin, USA; 4 Department of Population Health Sciences, University of Wisconsin, Madison, Wisconsin, USA

## Abstract

**Background:**

Overexpression of the *HER2 *proto-oncogene in human cancer cells has been associated with a poor prognosis, and survival improves with therapy targeting the *HER2 *gene. Animal studies and protein modeling suggest that the Ile655Val polymorphism located in the transmembrane domain of the HER2 protein might influence breast cancer development by altering the efficiency of homodimerization.

**Methods:**

To investigate this genetic polymorphism, incident cases of invasive breast cancer (*N *= 1,094) and population controls of a similar age (*N *= 976) were interviewed during 2001 to 2003 regarding their risk factors for breast cancer. By using DNA collected from buccal samples mailed by the participants, the *HER2 *Ile655Val polymorphism was evaluated with the Applied Biosystems allelic discrimination assay. Odds ratios (ORs) and 95% confidence intervals (95% CIs) were estimated by logistic regression adjusted for numerous breast cancer risk factors. Analysis was restricted to women with self-reported European descent.

**Results:**

Prevalence of the Val/Val genotype was 5.6% in cases and 7.1% in controls. In comparison with the Ile/Ile genotype, the Ile/Val genotype was not significantly associated with breast cancer risk (OR 0.97, 95% CI 0.79 to 1.18), whereas the Val/Val genotype was associated with a reduced risk (OR 0.63, 95% CI 0.42 to 0.92). This inverse association seemed strongest in older women (OR 0.51, 95% CI 0.29 to 0.89 for women aged more than 55 years), women without a family history of breast cancer (OR 0.54, 95% CI 0.35 to 0.84), postmenopausal women with greater body mass index (OR 0.43, 95% CI 0.20 to 0.91 for a body mass index of 25.3 kg/m^2 ^or more), and cases diagnosed with non-localized breast cancer (OR 0.49, 95% CI 0.26 to 0.90).

**Conclusion:**

Although results from our population-based case-control study show an inverse association between the *HER2 *Ile655Val polymorphism and risk of invasive breast cancer, most other studies of this single-nucleotide polymorphism suggest an overall null association. Any further study of this polymorphism should involve sample populations with complete risk factor information and sufficient power to evaluate gene-environment interactions between the *HER2 *polymorphism and factors such as age and family history of breast cancer.

## Introduction

The proto-oncogene human epidermal growth factor receptor 2 (*HER2*/*neu*, also called c-*erb*B-2) belongs to a family of receptors involved in the tyrosine kinase-mediated regulation of normal breast tissue growth and development [1]. *HER2 *amplification or overexpression is fairly common – present in 20 to 30% of human breast cancers – and is a significant predictor of response to therapy, prognosis, and overall survival [1]. *HER2 *is also a target for therapy. Antibody therapy with trastuzumab, which binds the extracellular portion of HER2, has been associated with improved patient outcomes including survival [2]. Because HER2 clearly has an important role in prognosis after a diagnosis of breast cancer, the gene encoding it is a natural target for investigation regarding polymorphisms that might indicate resistance or susceptibility for breast cancer development.

One single-nucleotide polymorphism (SNP) at codon 655 indicates a guanine-to-adenine substitution (Ile655Val) in the transmembrane domain-coding region of the *HER2 *gene [3]. This SNP has been evaluated in a variety of populations; studies show that the prevalence of the Val/Val genotype ranges from 3% to 7% in control women [4-6], although this genotype may be less common or unobserved in people with Asian or African descent [7-9].

Epidemiologic studies of the association between the Ile655Val polymorphism and breast cancer risk have generally shown null associations, with risk estimates below unity [4,5,10,11] and above unity [6,8,12-14]. Subgroup analysis in several studies suggested that, among women who were younger [7,8,14], physically inactive [7], had greater body mass [7], or had a positive family history of breast cancer [6,8], the Val/Val genotype was associated with an increased risk of breast cancer in comparison with the Ile/Ile genotype. Further study of this SNP has been supported because of the concern that subgroups of identifiable women might be especially susceptible to breast cancer [6,10]. In the present study we evaluated the association between the *HER2 *Ile655Val polymorphism and breast cancer risk in a population-based case-control study of midwestern United States women.

## Materials and methods

### Study subjects

As part of a continuing epidemiologic study, we recruited population-based cases of incident invasive breast cancer as well as community controls across Wisconsin in accordance with a protocol approved by the University of Wisconsin Health Sciences Human Subjects Committee. Invasive breast cancer cases (excluding carcinoma *in situ*) aged 20 to 69 years were identified though the Wisconsin statewide tumor registry. Controls were randomly sampled from driver's license files (ages 20 to 64 years) and Medicare beneficiary lists (ages 65 to 69 years); controls were frequency-matched in 5-year intervals to have a similar age distribution to that of the cases. All participants were required to have an available telephone number, and controls who self-reported a personal history of breast cancer were not eligible. Before April 2003, when changes in federal law affected the willingness of physicians to acknowledge their care of our eligible participants, physicians (identified on the tumor registry reports) were contacted before case enrollment to obtain information that might contraindicate study participation, such as dementia. All cases and controls were contacted by mail before receiving an interviewer's call. The 35-minute structured telephone interview elicited complete reproductive and menstrual histories, exogenous hormone use, smoking history, recent alcohol use and recreational physical activity, lifetime occupational and residential history, and exposure to indoor and outdoor chemicals. Information regarding the women's personal and family history of cancer was obtained at the end of the interview to maintain interviewer blinding. During April 2001 to January 2004, 77% of eligible cases (*N *= 1,884) and 70% of eligible controls (*N *= 2,146) participated in the telephone interview. The major reasons for nonparticipation were refusal (15% of cases, 23% of controls), death before the interview (2% of cases, 1% of controls), and inability to locate (3% of cases, 6% of controls). Before April 2003, physicians refused participation for 2% of cases.

At the conclusion of the telephone interview, all cases and controls were asked to provide a mouthwash rinse. Those agreeing were mailed a kit containing a 44 ml bottle of Scope mouthwash, consent forms, prepaid return mailing supplies, and other all materials needed for producing the sample. During April 2001 to January 2004, samples were obtained from 1,482 cases (79%) and 1,727 controls (81%). Genomic DNA was extracted by using the Gentra Systems DNA extraction reagents and protocol. DNA was resuspended in sterile water. Samples contained an average yield of 29.3 μg of DNA.

### Genotyping

The laboratory staff were blinded to the identity and disease status of the subjects. Samples were genotyped for the *HER2 *Ile655Val polymorphism with the Applied Biosystems allelic discrimination assay-by-design (no. 185078430). The primers and labeled oligonucleotide probes for this reaction were as follows: forward, 5'-CCTGACCCTGGCTTCCG-3' ; reverse, 5'-ACCAGCAGAATGCCAACCA-3' ; VIC probe (detects T), 5'-ACGTCCATCATCTC-3' ; FAM probe (detects C), 5'-CCATCGTCTCTGCG-3'. Samples were cycled with conditions recommended by ABI. Fluorescence was detected with the ABI 7700 and genotypes were called manually with the detection software for this instrument. Genotyping failed for 45 subjects (2%). For quality control, DNA from 79 subjects who had submitted two independent samples were genotyped; 100% (79 of 79) had identical genotypes for the two samples. *HER2 *genotype was obtained for the 1,098 invasive breast cancer cases and 991 controls with European descent who had mailed their mouthwash samples to study staff by 30 June 2003. Because of the small number of women with non-European descent (46 cases, 55 controls) and the low prevalence of the *HER2 *Val/Val genotype in Asian and African populations, these women were not genotyped.

### Statistical analysis

Only exposure status before an assigned reference date was used in this analysis. For cases, this was the date of breast cancer diagnosis. For comparability, control subjects were assigned a reference date corresponding to the average time from diagnosis to interview for the case group (about 1 year). The reference age was defined as the age at the reference date. Menopausal status was defined as postmenopausal if the subject reported natural menopause or bilateral oophorectomy before the reference date. Women reporting hysterectomy alone were classified as postmenopausal if their reference age was greater than or equal to the 90th centile of age at natural menopause for the control group (54 years for smokers and 56 years for nonsmokers). Menopausal status was considered to be unknown for women with hysterectomy without bilateral oophorectomy if their reference age was between 42 and 54 years (or 56 years for nonsmokers).

Adjusted odds ratios (ORs) and 95% confidence intervals (CIs) were obtained from multivariable conditional logistic regression models stratified on age. Covariates for the models were chosen by forward stepwise regression (*P*_entry _= 0.20, *P*_removal _= 0.30). After forward stepwise regression had been performed, covariates remaining in the model were: family history of breast cancer in a mother, daughter, or sister (yes, no, unknown), recent alcohol consumption (four categories), parity (four categories), menopausal status and age at menopause (four categories of age at menopause, premenopausal, unknown), hormone replacement therapy use (never, former, current), age at menarche (five categories of age, plus unknown), height at age 25 years (continuous), weight at age 18 years (continuous) and weight change since age 18 years (five categories). Covariates that did not remain in the final model included age at first birth, education, and income. Women with unknown recent alcohol consumption, hormone replacement therapy use, or height at age 25 years were not included in the analysis (4 cases, 15 controls), so that 1,094 cases and 976 controls remained in the analysis. Interactions with genotype in relation to breast cancer risk were evaluated by including a cross-product term in the regression model and measuring the change in the log-likelihood.

## Results

Breast cancer cases were more likely than controls to report a positive family history of breast cancer, to drink modest amounts of alcohol, to have lower parity, to report menopause at later ages, to have a younger age at menarche, and to report taller adult height (Table 1). Cases in this *HER2 *analysis were slightly less likely to have non-localized breast cancer at diagnosis; 32% of cases who contributed buccal samples that were included in the *HER2 *analysis had regional or distant-staged disease at diagnosis, whereas 37% of cases who refused to contribute a sample had non-localized disease (*N *= 356, *P *= 0.07 by Fisher's exact test). Participants in this analysis were similar to nonparticipants in body mass index (*P *= 0.14 for cases, *P *= 0.29 for controls by *t*-test; *N *= 442 nonparticipant controls) and family history of breast cancer (*P *= 0.27 for cases, *P *= 0.36 for controls by Fisher's exact test), although control participants were somewhat older (55 versus 53 years, *P *= 0.02 by *t*-test) and more likely to have attended college than nonparticipant controls (56% versus 50%, *P *= 0.05 by Fisher's exact test). Among cases, participants in this analysis did not differ significantly from nonparticipants in age (54 versus 53 years, *P *= 0.73) but were slightly more likely to have attended college (57% versus 52%, *P *= 0.09).

The Ile allele frequency was similar for cases and controls (cases 76.3%, 95% CI 74.5 to 78.1%; controls 74.7%, 95% CI 72.8 to 76.6%), and the Val allele frequency was about 25% (cases 23.7%, 95% CI 21.9 to 25.5%; controls 25.3%, 95% CI 23.4 to 27.2%); 58.2% of cases and 56.5% of controls were homozygous for the Ile allele, 36.2% of cases and 36.5% of controls were heterozygous, and 5.6% of cases and 7.1% of controls were homozygous for the Val allele (Table 2). Both the case group (*P *= 0.96) and the control group (*P *= 0.28) were consistent with Hardy-Weinberg equilibrium.

After multivariable adjustment, the combined Ile/Val and Val/Val genotypes were not significantly associated with a risk of breast cancer relative to two copies of the Ile allele (OR 0.90, 95% CI 0.75 to 1.09; Table 2). The presence of two copies of the Val allele was associated with a 37% reduced risk of breast cancer compared with the Ile/Ile genotype (OR 0.63, 95% CI 0.42 to 0.92). Whereas this inverse association was suggested for cases diagnosed with localized breast cancer (OR 0.69, 95% CI 0.45 to 1.06), the OR was significantly reduced for cases diagnosed with regional or distant metastasis (OR 0.49, 95% CI 0.26 to 0.90).

Although no interactions between the *HER2 *polymorphism and common risk factors were statistically significant, the inverse association with breast cancer risk was strongest in some subgroups (Table 3). In particular, ORs were significantly reduced for women at older ages (more than 55 years), without a family history of breast cancer, with older age at menarche, currently using postmenopausal hormones, with greater recent body mass index, and women with greater weight gain since age 18 years. In addition, we could not find evidence to support heterogeneity in the association between the *HER2 *Ile655Val polymorphism and breast cancer risk according to recent physical activity (*P *= 0.45), cigarette smoking status (*P *= 0.66), adult height (*P *= 0.78), recent alcohol intake (*P *= 0.83), parity (*P *= 0.81), or age at menopause (*P *= 0.41) (data not shown).

## Discussion

We observed a 40 to 50% decreased risk of breast cancer associated with the inheritance of two *HER2 *valine alleles at codon 655 for some subgroups of women, including women older than 55 years of age and women without a family history of breast cancer. Three other studies – one study of Asian women [11] and two studies of women with European descent [4,5,10] – have also reported decreased risk estimates of breast cancer associated with inheritance of the *HER2 *Val allele, although the estimates from these three other studies were not statistically significant.

Our null results for younger women and women with a positive family history of breast cancer do not concur with findings by Montgomery and colleagues [14], which showed a threefold increased risk among Australian women less than 40 years of age. Wang-Gohrke and Chang-Claude [6] reported a twofold increased risk among German Caucasians with a first-degree family history of breast cancer. Similarly, Millikan and colleagues [8] reported a twofold increased risk of breast cancer associated with the Val/Val or Val/Ile genotype (compared with the Ile/Ile genotype) among women living in North Carolina (United States) who were both less than 45 years of age and reported a positive family history of breast cancer (OR 2.3, 95% CI 1.0 to 5.3). We were limited in our ability to examine the *HER2 *polymorphism in younger women because of small numbers. Only 4 controls and 12 cases in our study were 45 years of age or younger, reported a positive family history of breast cancer, and also had the Val/Val or Val/Ile genotype (OR 1.44, 95% CI 0.21 to 9.79, with Ile/Ile as the reference category; data not shown).

The first study of the *HER2 *Ile655Val polymorphism in relation to breast cancer risk found a very high risk (OR 14.1, 95% CI 1.8 to 113.4) of the Val/Val versus Ile/Ile genotype [7]. In that study, the Val/Val genotype was detected in only 11 cases and 1 control. Risk estimates in subsequent studies have been much more modest, ranging from 0.3 to 2.8, and our results clearly fall within this (wide) range. Although risk estimates have suggested both inverse and positive associations with breast cancer risk, prevalence of the Val/Val genotype has consistently been 3 to 8% in breast cancer cases and 3 to 7% in controls in women with European descent. Allele frequencies for case and control women corresponding to the Val/Val genotype in our study are very similar to frequencies reported in three other studies of white women in North Carolina, southeast England, and Germany – ranging from 23% to 25% – and slightly higher than frequencies for control women in two other studies conducted in Australia and New York City (18.7% and 16%, respectively) [5,6,8,13,14].

Most studies of the *HER2 *Ile655Val polymorphism have used a case-control design. Only one study population was a prospective cohort [12]. Two other published reports used a kin-cohort approach [15,16]. Using this novel design with a study of 1,560 volunteers living in Washington DC and Israel, Rutter and colleagues [16] reported that the *HER2 *valine allele might be associated with a twofold to eightfold increased risk of breast cancer. As with the Millikan study [8], these increased risks were confined to younger women with a family history of breast cancer.

Many studies of the *HER2 *Ile655Val polymorphism had insufficient power to evaluate interactions between the SNP and subgroups according to risk factors such as age and family history of breast cancer. Limited power is a common problem in studies of genetic polymorphisms. Sample size for only one other study was larger than the case and control enrollment in our own study [8]. Prevalence of the Ile655Val polymorphism clearly varies according to racial descent – it is rare or unobserved in Asian and African populations [9,17] – further limiting statistical power to evaluate the significance or relevance of this SNP in different populations. Stratified analysis of the *HER2 *Ile655Val genotype according to racial descent is warranted.

Potential limitations might have influenced our findings. Although participation in our study was excellent for a population-based case-control study, certain subgroups might have been under-represented because participation probably declines with increasing age, decreasing attained education, and other factors. However, genetic inheritance with the *HER2 *gene is probably not confounded with the variables that might influence a woman's participation in our epidemiologic study [18]. The distribution of the *HER2 *polymorphism in our case and control groups was consistent with Hardy-Weinberg equilibrium, which suggests that any genotyping errors were not substantial. Duplicate genotyping of 79 samples was also reassuring, achieving 100% concordance.

The mechanism through which this SNP might influence breast cancer risk is unclear, although studies in transgenic mice have demonstrated that activation or overexpression of the *HER2 *gene leads to the development of mammary adenocarcinomas [19-21]. The transmembrane domain of the HER2 protein might be especially important, given the discovery of an activating mutation in codon 664 in the rat [22-25]. In humans, the Ile655Val amino acid substitution might alter the formation of active HER2 dimers, which would then alter the activity of the protein [26].

## Conclusion

These data from our sample population of white women from the midwestern United States suggest that the Val/Val genotype of the *HER2 *Ile655Val polymorphism is associated with a reduced risk of breast cancer in comparison with the Ile/Ile genotype for some women. Although the sample size in our study was relatively large compared with other studies published so far, the inconsistency of the findings across all studies argues against a strong relation with breast cancer risk. Future large studies of the *HER2 *polymorphism might clarify this putative gene-environment interaction. However, given the promise of innovative and more comprehensive approaches to genomic and proteomic studies of breast cancer risk, focusing on this SNP without consideration of the role of other genes and polymorphisms may not be warranted.

## Abbreviations

CI = confidence interval; OR = odds ratio; SNP = single-nucleotide polymorphism.

## Competing interests

The author(s) declare that they have no competing interests.

## Authors' contributions

SEN performed the processing and genotyping of the samples and drafted the manuscript. MNG and ATD jointly conceived of and designed the study, obtained funding, and drafted the manuscript. JMH performed the statistical analysis and assisted with manuscript preparation. All authors read and approved the final manuscript.

## Figures and Tables

**Table 1:** Characteristics of invasive breast cancer cases and population controls, Wisconsin, 2001 to 2003

Characteristic	Cases (*N *= 1,094)		Controls (*N *= 976)		OR^†^	95% CI^†^
	*n*	%	*n*	%*		
Family history of breast cancer						
Absent	855	78.2	829	85.3	1 (Reference)	
Present	231	21.1	132	13.1	1.69	1.33–2.16
Unknown	8	0.7	15	1.7	0.44	0.18–1.05
Recent alcohol consumption						
None	153	14.0	166	16.8	1 (Reference)	
1 drink/week	424	38.8	418	42.5	1.10	0.84–1.45
2–6 drinks/week	385	35.2	270	28.2	1.57	1.19–2.09
7 or more drinks/week	132	12.1	122	12.4	1.23	0.87–1.74
Parity						
0–1	262	23.9	217	23.4	1 (Reference)	
2	392	35.8	275	29.7	1.22	0.95–1.55
3	253	23.1	209	21.3	0.99	0.76–1.30
4 or more	187	17.1	275	25.6	0.58	0.44–0.77
Menopausal status						
Postmenopausal	597	54.6	592	55.1	1 (Reference)	
Premenopausal	415	37.9	313	36.8	1.25	0.92–1.70
Unknown	82	7.5	71	8.1	1.06	0.71–1.58
Age at menopause (years)^‡^						
<45	138	23.1	175	30.1	1 (Reference)	
45–49	113	18.9	127	21.4	1.15	0.80–1.65
50–54	205	34.3	172	29.0	1.61	1.16–2.23
55+	68	11.4	65	10.1	1.42	0.91–2.20
Unknown	73	12.2	53	9.4	1.76	1.14–2.77
Body mass index (kg/m^2^)^‡^						
<22.6	129	21.6	135	23.1	1 (Reference)	
22.6–25.2	144	24.1	136	22.8	1.18	0.82–1.69
25.3–28.9	166	27.8	161	26.8	1.09	0.77–1.54
29.0+	153	25.6	156	26.6	0.97	0.69–1.38
Weight change since age 18 (kg)^‡^						
Lost 5 or more	14	2.3	20	3.5	0.92	0.42–2.03
Lost 5 to gained 4	95	15.9	109	18.9	1 (Reference)	
Gained 5 to 11	148	24.8	140	23.3	1.42	0.97–2.09
Gained 12 to 21	179	30.0	172	28.7	1.30	0.90–1.88
Gained 22 or more	154	25.8	143	24.3	1.32	0.91–1.93
HRT use^‡^						
Never	184	30.8	233	37.9	1 (Reference)	
Former	45	7.5	77	12.6	0.65	0.42–1.01
Current	368	61.6	282	49.4	1.49	1.14–1.95
Age at menarche (years)						
< 12	228	20.8	176	18.1	1 (Reference)	
12	282	25.8	253	26.4	0.83	0.64–1.09
13	297	27.1	244	24.4	0.94	0.72–1.23
14	183	16.7	172	17.6	0.81	0.61–1.10
15+	98	9.0	119	12.4	0.63	0.45–0.89
Height at age 25 (m)						
< 1.60	198	18.1	218	21.9	1 (Reference)	
1.60–1.64	295	27.0	263	27.0	1.25	0.96–1.62
1.65–1.67	299	27.3	264	26.9	1.26	0.97–1.64
1.68+	302	27.6	231	24.1	1.47	1.12–1.92

*Control percentages are age-adjusted to the distribution of cases; ^†^logistic regression models conditional on age; ^‡^among postmenopausal women. CI, confidence interval; HRT, hormone replacement therapy; OR, odds ratio.

**Table 2:** Risk of invasive breast cancer according to the *HER2 *Ile655Val polymorphism

Polymorphism	Cases		Controls		OR*	95% CI*	OR^†^	95% CI^†^
	*N*	%	*N*	%				
All subjects								
Ile/Ile	637	58.2	551	56.5	1 (Reference)		1 (Reference)	
Ile/Val or Val/Val	457	41.8	425	43.5	0.92	0.76–1.10	0.90	0.75–1.09
Ile/Val	396	36.2	356	36.5	0.96	0.79–1.16	0.97	0.79–1.18
Val/Val	61	5.6	69	7.1	0.71	0.49–1.04	0.63	0.42–0.92
Localized disease^‡^								
Ile/Ile	425	58.5	551	56.5	1 (Reference)		1 (Reference)	
Ile/Val or Val/Val	301	41.5	425	43.5	0.91	0.74–1.12	0.90	0.73–1.12
Ile/Val	257	35.4	356	36.5	0.94	0.76–1.16	0.95	0.76–1.19
Val/Val	44	6.1	69	7.1	0.78	0.52–1.18	0.69	0.45–1.06
Regional or distant metastasis^‡^								
Ile/Ile	195	57.5	551	56.5	1 (Reference)		1 (Reference)	
Ile/Val or Val/Val	144	42.5	425	43.5	0.96	0.74–1.25	0.96	0.73–1.27
Ile/Val	128	37.8	356	36.5	1.03	0.79–1.36	1.08	0.81–1.44
Val/Val	16	4.7	69	7.1	0.60	0.34–1.09	0.49	0.26–0.90

*Logistic regression models conditional on age; ^†^logistic regression models conditional on age and adjusted for family history of breast cancer, recent alcohol consumption, parity, menopausal status, age at menopause, hormone replacement therapy use, age at menarche, height at age 25 years, weight at age 18 years, and weight change since age 18 years; ^‡^for cases at diagnosis. CI, confidence interval; OR, odds ratio.

**Table 3:** Risk of invasive breast cancer according to the *HER2 *Ile655Val polymorphism and common risk factors

Risk factor*	*HER2 *polymorphism				
	
	Ile/Ile, cases/controls	Val/Val, cases/controls	Ile/Ile, OR^†^, 95% CI	Val/Val, OR^† ^(95% CI)	*P*^‡^
Age (years)					0.29
<55	317/256	32/29	1 (reference)	0.78 (0.44–1.37)	
55+	320/295	29/40	1 (reference)	0.51 (0.29–0.89)	
Family history of breast cancer					0.24
None	505/461	41/59	1 (reference)	0.54 (0.35–0.84)	
Any	128/79	19/10	1 (reference)	0.92 (0.32–2.62)	
Age at menarche (years)					0.14
<13	288/249	32/35	1 (reference)	0.88 (0.50–1.54)	
≥ 13	346/297	29/33	1 (reference)	0.47 (0.27–0.84)	
HRT use^§^					0.19
Never/former	149/169	12/10	1 (reference)	1.24 (0.46–3.39)	
Current	212/163	18/23	1 (reference)	0.46 (0.22–0.97)	
Recent body mass index^§^					0.07
<25.3 kg/m^2^	159/166	14/10	1 (reference)	1.01 (0.39–2.64)	
≥ 25.3 kg/m^2^	196/163	14/25	1 (reference)	0.43 (0.20–0.91)	
Weight change since age 18 years (kg)^§^					0.12
Lost 5 to gained 11	142/148	14/11	1 (reference)	1.04 (0.41–2.67)	
Gained 12 or more	204/164	14/22	1 (reference)	0.44 (0.20–0.98)	

*Risk factor cut-points based on the approximate median values for the controls; ^†^logistic regression models conditional on age and, as appropriate, adjusted for family history of breast cancer, recent alcohol consumption, parity, menopausal status, age at menopause, hormone replacement therapy use, age at menarche, height at age 25 years, weight at age 18 years, and weight change since age 18 years; ^‡^*P *interaction using the likelihood ratio test and assuming a multiplicative model (risk factors parameterized as dichotomous variables as shown in the table for purposes of the interaction tests); ^§ ^postmenopausal women only. CI, confidence interval; HRT, hormone replacement therapy; OR, odds ratio.
